# The Role of Fas/Fas Ligand System in the Pathogenesis of Liver Cirrhosis and Hepatocellular Carcinoma

**DOI:** 10.5812/hepatmon.6132

**Published:** 2012-11-03

**Authors:** Olfat Hammam, Ola Mahmoud, Manal Zahran, Sohair Aly, Karim Hosny, Amira Helmy, Amgad Anas

**Affiliations:** 1 Departments of Pathology, Theodor Bilharz Research Institute, Cairo, Egypt; 2 Departments of Hematology, Theodor Bilharz Research Institute, Cairo, Egypt; 3 Malaysia and Medicinal Chemistry Department, Advanced Dental and Medical institute, IPPT, USM, NRC, Cairo, Egypt; 4 Surgical Department, Kasr El Aini Hospital, Cairo, Egypt; 5 Departments of Electron Microscopy, Theodor Bilharz Research Institute, Cairo, Egypt; 6 Departments of Gastroenterology, Theodor Bilharz Research Institute, Cairo, Egypt

**Keywords:** SfaS Protein, Liver Cirrhosis, Carcinoma, Hepatocellular

## Abstract

**Background:**

The Fas receptor/ligand system including soluble forms is the most important apoptotic initiator in the liver. Dysregulation of this pathway may contribute to abnormal cell proliferation and cell death and is regarded as one of the mechanisms preventing the immune system from rejecting the tumor cells.

**Objectives:**

To analyze the role of Fas system Fas/ Fas ligand (Fas/ FasL) in the multi-step process of hepatic fibrosis/carcinogenesis, and to use of the serum markers as possible candidate biomarkers for early detection of hepatocellular carcinoma (HCC).

**Patients and Methods:**

Ninety patients were enrolled: 30 cases of chronic hepatitis C (CHC) without cirrhosis, 30 cases of CHC with liver cirrhosis, and 30 cases of HCC and hepatitis V virus (HCV) infection. Ten wedge liver biopsies, taken during laparoscopic cholecystectomy, were served as normal controls. Serum soluble Fas (sFas) levels were measured using ELISA technique; Fas and FasL proteins were detected in hepatic tissue by indirect Immuno-histochemical technique (IHC); electron microscopic (EM) and immune electron microscopic examinations were performed for detection of Fas expression on lymphocytes.

**Results:**

Hepatic expression of both Fas and FasL as well as expression of Fas on separated lymphocytes were significantly increased in the diseased groups (P < 0. 01) compared to the control specimens. The highest expression was noticed in CHC specimens, particularly with the necro-inflammatory activity and advancement of the fibrosis. The sFas in cirrhotic patients and HCC were significantly higher than that in normal controls and CHC without cirrhosis group (P < 0.01).

**Conclusions:**

Apoptosis and the Fas system were significantly involved in the process of converting liver cirrhosis into hepatocellular carcinoma. Down-regulation of Fas expression, up regulation of FasL expression in hepatocytes, and elevation of serum sFas levels were important in tumor evasion from immune surveillance, and in hepatic carcinogenesis.

## 1. Background

Hepatitis C virus (HCV) infects more than 170 million people worldwide representing a major threat to global public health. Most of infected patients are chronically infected and at risk of life-threatening complications such as liver cirrhosis and hepatocellular carcinoma (HCC) (1). Immuno-phenotyping of intrahepatic infiltrating inflammatory cells in chronic hepatitis C patients showed a predominance of T lymphocytes cells, with a significant proportion of CD4+ and CD8+ cells, suggesting that the host immune system is involved in liver disease pathogenesis (2). Hepatocyte aberrations, accumulation of chromosomal damage, and possibly initiation of hepatic carcinogenesis are thought to initiate by continued viral replication and persistent attempt through a less-than-optimal immune response to eliminate HCV-infected cells (3). The genetically altered cells that have undergone malignant transformation can be cleared efficiently by the immune system charged with the function of cancer surveillance. Tumor cells survive, expand their populations, and metastasize by developing strategies to avoid clearance by the immune system (4). An attractive insight into mechanism of tumor escape from immune clearance has been provided by the identification of the “Death factors”, including Fas/Fas Ligand (Fas/FasL) as an important regulator of both apoptosis and immune function (5). Fas is a type I membrane protein which belongs to tumor necrosis factor (TNF) receptor family, whereas the FasL is a member of TNF family. The FasL binding to its Fas receptor triggers a cascade of well-characterized intracellular signaling events that ends in cell death by apoptosis (6). Fas receptors are widely expressed in normal and diseased tissues. It has been implicated in tumor progression of several cancers (7, 8). FasL is expressed mainly in cytotoxic T lymphocytes (CTL) (9), immune privileged sites (10), and in various tumors where specific cytotoxic T cell clones are produced (11). FasL over-expression was found to relate to advanced stages of many tumors (8, 12). Apoptosis is tightly regulated throughout a variety of mechanisms, one of which is postulated to be the production of sFas, an antagonistic decay protein similar to Fas, lacking exceptionally the trans-membrane domain. It normally binds to FasL and blocks the signaling of membrane-bound form of Fas. Elaboration of sFas by tumor cells by alternative mRNA splicing may contribute to resistance to Fas-mediated apoptosis (13).

## 2. Objectives

This study was designed to assess the hepatic tissue expression of Fas/FasL and Fas expression on T lymphocytes using electron microscopic (EM) and immuno-electron microscopic (IEM) examinations as well as the circulating serum levels of sFas in chronic hepatitis C (CHC) liver disease; to analyze the role of these factors in the multi-step process of fibrosis/carcinogenesis; and the possible use of serum markers as possible candidate biomarkers for early detection of HCC.

## 3. Patients and Methods

This study enrolled 90 chronic HCV Patients (54 males and 36 females, aging between 24-66 years with a mean age of 48.32 ± 7.65 years) admitted to the Hepatology and Gastroenterology Department at Theodor Bilharz Research Institute (TBRI). Tumor specimens were taken by endoscopy and surgical specimens were obtained from partial hepatectomies at Kasr Al Aini Hospital. They included 30 cases of CHC without hepatic cirrhosis, 30 cases of CHC with hepatic cirrhosis, and 30 cases with HCC. Patients with liver disease of other etiologies were excluded. The diagnosis of cirrhotic patients was based on clinical history, clinical examinations, laboratory findings, gastroscopy, and ultrasonography. In addition, ten control wedge liver biopsies were taken from age- and sex-matched individuals subjected to laparoscopic cholecystectomy (seven males and three females, aging between 31-45 years with a mean age of 42.21 ± 4.54 years). Written informed consent was obtained from all participants and the TBRI local ethical committee approved the study.

### 3.1. Sampling

Blood samples of 8 mL were collected into sterile endotoxin-free vacuum blood collection tubes on potassium EDTA. The peripheral blood mononuclear cell (PBMNC) layer was separated using Ficoll Hypaque (Seromid Biochrom, Berlin, Germany) density gradient centrifugation and washed 3 times with Hank’s balanced salt solution (HBSS) without Ca2+ and Mg2+ ions (14). These cells were prepared for EM examination using agarose cell-block technique in addition to IEM examination. Other blood samples of 2 mL were withdrawn into plain tube and centrifuged shortly after clot formation. All samples were stored at −70°C in aliquots and used for analysis of sFas. Formalin-fixed and paraffin-embedded tissues from tumor samples were used for immunohistochemical (IHC) analysis of Fas and FasL. Liver biopsies were analyzed according to a histological METAVIR (15) scoring system using two separate scores; one for necro-inflammatory activity (A) grading (A1: minimal activity, A2: moderate activity, A3: severe activity), and the other for fibrosis (F) staging ( scoring from F0-F4; F1 to F2: significant fibrosis, F3 to F4: advanced fibrosis). Routine laboratory investigations were performed, including complete haemogram using automated haemogram (ACT Differential, Beckman -Coulter, France). Liver function tests were carried out using commercially available kits. Circulating anti-HCV antibodies were detected using Murex enzyme immunoassay kit (Murex anti-HCV, Version V; Murex Diagnostics; Dartford, England). The presence of HCV-RNA in patients’ sera was detected by real-time polymerase chain reaction using the Amplicor test (Roche Diagnostic Systems; Meylan, France).

### 3.2. Immunohistochemical analysis

Avidin biotin complex (ABC) immune peroxidase technique was used to perform the immune histochemical reaction according to Hsu and Raine (16). Anti-human Fas and FasL on paraffin sections was used; dewaxed in xylene and hydrated in descending grades of ethanol; treated with 3% hydrogen peroxide in 100% methanol for 20 min to quench the endogenous peroxidase activity; and microwaved at high power (700W) for 15 minutes in citrate buffer (pH 6.0) for antigen retrieval. Sections were then incubated overnight at 4°C with the following primary monoclonal antibodies: human anti-Fas and anti-Fas ligand (purchased from Santa Cruz Biotechnology Inc.; Santa Cruz, USA) diluted at 1:100, 1:150, respectively in Phosphate buffer saline (PBS). Next day, after thorough washing in PBS, the sections were incubated with streptavidin-biotin-peroxidase preformed complex using a peroxidase/DAB (3, 3’-diaminobenzidine) enzymatic reaction. The staining was completed by 5-10 minutes resulted in a brown-colored precipitate at the antigen sites, counterstained in Mayer’s hematoxylin to visualize the cell nuclei and mounted by a cover slip using DPX mounting medium. Positive and negative control slides for each marker were included within each session. As a negative control, liver tissue section was processed in the above-mentioned sequences but the omission of the primary antibody and PBS were replaced. The scoring of Fas/FasL in liver tissue was based on intensity and extensiveness (by percentage of population) of the positively stained cells. Both parameters were scored on 0-3 scale basis as follows:

1. Intensity: 0 = negative staining (-), 1 = weakly positive staining (+), 2 = moderately positive staining (++), and 3 = strongly positive staining (+++),according to Chen et al.(17);

2. Extensiveness range: 0 = negative, 1 = positive staining in < 10% of cells, 2 = positive in 10%-50% of cells, and 3 = positive in > 50% of cells, according to Itoi et al. (18). Liver sections were examined by Zeiss light microscopy at power X400 for both markers; the number of positively stained cells with the highest expression was semi-quantitatively recorded within ten successive fields per section and the final value represents the mean. Zero percentage was given to unstained sections.

### 3.3. Measurements of Serum sFas Levels

Serum Fas (sFas) levels were assayed using a sandwich enzyme-linked immune-sorbent assay kit (ELISA) (Biosource International, Camarillo, California, USA) according to the manufacturer’s instructions. The optical density was measured at 450 nm wavelength using an ELISA reader (PR 3100 TSC Microplate Reader Bio-Rad). The concentration of sFas in serum samples was determined from the standard curve. All assays were conducted in duplicates, and the mean concentration of sFas was calculated. Electron microscopic and immuno-electron microscopic (IEM) examinations of peripheral blood mononuclear cells (PBMNCs) were done using agarose cell-block technique (19). Solidified agarose blocks of separated cells were refixed in buffered 4% glutaraldehyde for 1 hour, and then postfixed in 2% osmic acid for 1 hour, dehydrated in ascending alcohol, and infiltrated and embedded in epoxy resin. Ultrathin sections were performed using Leica ultramicrotome (Leica Microsystems GmbH, Ernst-Leitz-Strasse, Germany). The sections were stained with uranyl acetate and lead citrate, and examined using Philips Microscopic electron microscope 208 S (Eindhoven, The Netherlands). IEM examination was performed on other part of separated cells using rabbit polyclonal anti-Fas antibodies (Maixin-Bio, Fuzhou, China) as above method of immunohistochemical analysis, and then processed into agarose cell-block and prepared for EM examination as above.

### 3.4. Statistical Analysis

Statistical evaluation of results was done using SPSS computer program (version 12 windows). Results were expressed as mean ± standard deviation (SD) or number (%). Comparison between the mean values of different parameters in the different groups were performed using one way analysis of variance (ANOVA) with post hoc using the least significant difference. Correlation between parameters was performed using Spearman’s rank correlation coefficient (r) (20). P value < 0.05 was considered significant and < 0.01 was considered highly significant.

## 4. Results

### 4.1. Hepatic Expression of Fas/ FasL Antigen

**Figure 1:**
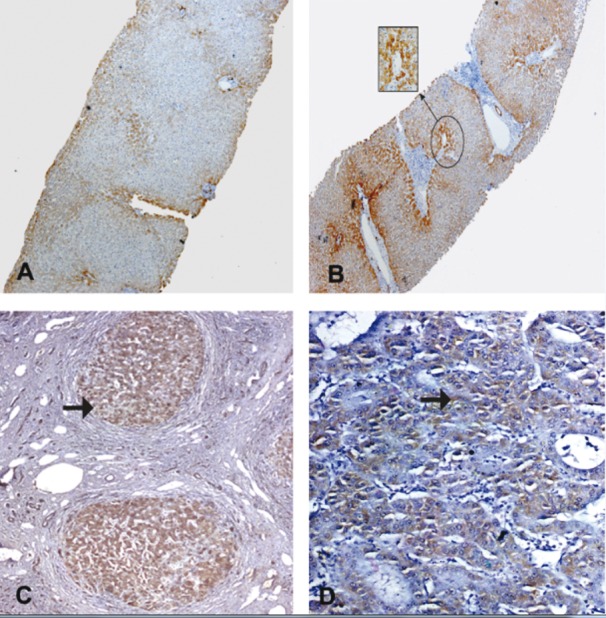
Immune Staining for Fas Monoclonal Antibody A) normal hepatocyte from a control case, showing faint expression of Fas as membranous ± cytoplasmic stain in the hepatocytes (IHC, DAB, X100). B) A case of CHC without cirrhosis, A1F1,showing moderate expression of Fas as membranous ± cytoplasmic stain in the hepatocytes, bile ducts, and lymphocytes at periportal area (IHC, DAB, X100). C) A case of CHC with cirrhosis, A2F3, showing cirrhotic nodule with moderate to marked expression of Fas as membranous ± cytoplasmic stain in the hepatocytes, bile ducts, and lymphocytes at periportal area (arrow)( IHC, DAB, X 100). D) A case of moderately differentiated HCC, showing mildly expressed Fas in the cytoplasm of hepatocytes (arrow) (IHC, DAB, X200).

**Figure 2:**
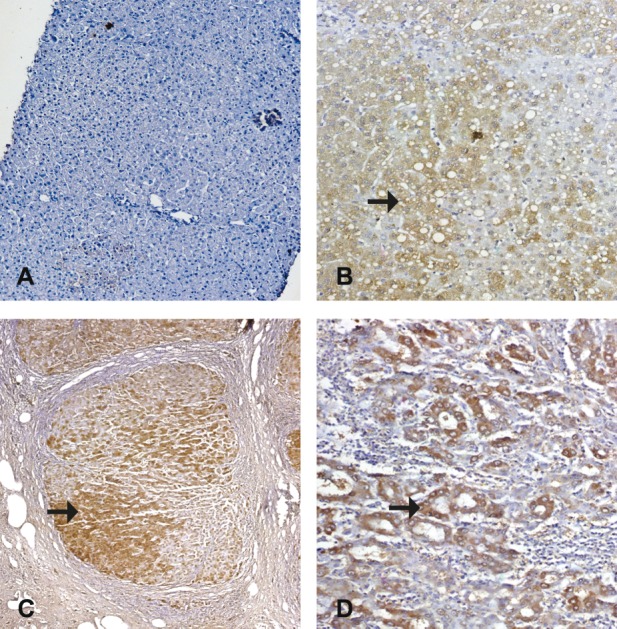
Immune Staining for Fas Ligand Monoclonal Antibody A) A normal hepatocyte in a control case, showing negative expression of Fas ligand (IHC, DAB, X100), B) A case of CHC without cirrhosis, A1F1, showing moderate expression of Fas ligand as cytoplasmic stain in the hepatocytes (IHC, DAB, X200), C) A case of CHC with cirrhosis, showing cirrhotic nodule with moderate expression of Fas ligand as cytoplasmic stain in the hepatocytes. (IHC, DAB, X100), D) A case of moderately differentiated HCC, showing moderately expressed Fas ligand in the cytoplasm of hepatocytes (IHC, DAB, X200).

Fas/FasL protein was observed in the membrane ± cytoplasm of hepatocytes with occasional perinuclear staining. In the current study, normal liver specimens showed faint Fas protein expression and no detectable FasL protein expression. Both antigens were significantly increased in the diseased groups (P < 0. 01) compared to the control specimens (Table 1, 2, and 3). In CHC, Fas was detected among infiltrating lymphocytes at the advancing edges of piecemeal necrosis (interface hepatitis). Moreover, FasL protein was expressed dominantly in the infiltrating mononuclear cells in portal area and hepatic sinusoids. Our data (Table 1, 2, and 3) showed a progressive Fas/FasL increase from chronic hepatitis (CH) to cirrhosis. Hepatic Fas expression was significantly decreased when comparing HCC with cirrhotic patients (P < 0.5). On the other hand, FasL expression in HCC group showed a non-significant difference compared to CHC with cirrhosis group. In CHC, apoptotic Fas/FasL protein expression was significantly increased with the necro-inflammatory activity and the advancement of fibrosis, according to METAVIR scoring systems (Table 4 and 5) (Figures 1 and 2).

**Table 1:** Mean ± SD of the Studied Parameters in All Groups

	Control, (n = 10), Mean ± SD	CHC Without Cirrhosis, (n = 30), Mean ± SD	CHC With Cirrhosis, (n = 30), Mean ± SD	HCC, (n = 30), Mean ± SD
**Tissue Fas, within 10 successive microscopic fields (× 400)/ section (mean percentage) + ve cells ± S.D**	1.8 ± 0.8	25.0 ± 5.74 a	61.36 ± 4.69 a, b	27.3 ± 5.6 a, c
**Tissue FasL, within 10 successive microscopic fields (× 400)/ section (mean percentage)+ ve cells ± SD**	00 ± 00	33.46 ± 11.91 a	49.83 ± 8.20 a, b	45.32 ± 6.4 a, b
**sFas, pg/ml**	165.5 ± 45.6	238.27 ± 135.29 a	814.94 ± 362 a, b	762.18 ± 437 a, b

Abbreviations: CHC, chronic hepatitis C; HCC, hepatocellular carcinoma.

^a^P Statistically significant from control group (P < 0.01).

^b^P Statistically significant from CHC without cirrhosis group (P < 0.01).

^c^P Statistically significant from cirrhosis group (P < 0.01).

**Table 2:** Tissue Expression of Fas Immune Staining in Liver Tissue of Different Studied Cases

Histopathological Diagnosis, No.	Positive Cases, No. (%)	Range, No.			Intensity, %		
		< 10%	10% - 50%	> 50%	+	+ +	+ + +
**Controls, 10**	2 (20)	2	-	-	2	-	-
**CHC without cirrhosis, 30**	10 (33.3) a	2	8	-	3	7	-
**CHC with cirrhosis, 30**	16 (54.3) a, b	5	6	5	4	8	4
**HCC, 30**	13(43.3) a, b, c	1	5	7	-	6	7

Abbreviations: CHC, chronic hepatitis C; HCC, hepatocellular carcinoma.

^a^P < 0.01 significant difference relative to control group.

^b^P < 0.05 significant difference relative to CHC without cirrhosis.

^c^P < 0.05 significant difference relative to CHC with cirrhosis.

**Table 3:** Tissue Expression of FasL Immune Staining in Liver Tissue of Different Studied Cases

Histopathologic Diagnosis, No.	Positive Cases, No. (%)	Range, No.			Intensity, %		
		< 10%	10% - 50%	> 50%	+	+ +	+ + +
**Controls, 10**	0 (0)	-	-	-	-	-	-
**CHC without cirrhosis, 30**	13 (43.3) a	3	10	-	8	5	-
**CHC with cirrhosis, 30**	17 (56.6) a, b	6	6	5	4	8	5
**HCC, 30**	16 (53.3) a, c	2	7	7	-	7	9

Abbreviations: CHC, chronic hepatitis C; HCC, hepatocellular carcinoma.

^a^P < 0.01 significant difference relative to control group.

^b^P < 0.05 significant difference relative to CHC without cirrhosis.

^c^P < 0.05 significant difference relative to CHC with cirrhosis.

**Table 4:** Mean ± SD of the Studied Parameters According to METAVIR Activity Scoring System in Chronic Hepatitis C With or Without Cirrhosis Groups

	A1 (n = 28)	A2 (n = 18)	A3 (n = 14)
**Tissue Fas, within 10 successive microscopic fields (× 400)/ section (mean percentage) + ve cells ± S.D**	23.57 ± 5.75	27.500 ± 3.54	43.00 ± 4.43 a, b
**Tissue FasL, within 10 successive microscopic fields (× 400)/ section (mean percentage) + ve cells ± S.D**	17.14 ± 4.23	20.00 ± 7.32	45.40 ± 6.42 a, b
**s Fas pg/ml**	210.4 ± 23.2	395.8 ± 41.4 a	447.5 ± 51.7 a, b

^a^P Statistically significant from A1 group (P < 0. 01).

^b^P Statistically significant from A2 group (P < 0.01).

**Table 5:** Mean ± SD of the Studied Parameters According to METAVIR Fibrosis Scoring System in Chronic Hepatitis C With or Without Cirrhosis Groups

	F1, (n = 24)	F2, (n = 15)	F3, (n = 14)	F4, (n = 7)
**Tissue Fas, within 10 successive microscopic fields (× 400)/ section (mean percentage) + ve cells ± S.D**	17.40 ± 6.21	19.759 ± 9.76	23.04 ± 2.08 a	33.28 ± 5.16 a, b, c
**Tissue FasL, within 10 successive microscopic fields (× 400)/ section (mean percentage) + ve cells ± S.D**	15.160 ± 5.02	18.75 ± 1.30	21.41 ± 2.28 a, b	28.42 ± 6.06 a, b
**s Fas pg/ml**	200.4 ± 47.1	250.3 ± 33.6	260.4 ± 54.7 a	340.3 ± 65.4 a, b, c

^a^P Statistically significant from F1 group (P < 0. 01).

^b^P Statistically significant from F2 group (P < 0.01).

^c^P Statistically significant from F3 group (P < 0.01).

### 4.2. Serum levels of sFas

Concentrations of studied parameter in CHC, HCC patients, and normal controls are shown in Table 1, 4, and 5. The serum levels of sFas in both HCC and cirrhotic patients were significantly higher than that in normal controls and CHC without cirrhosis (P < 0.01), but there was no significant difference between cirrhotic and HCC patients.

Correlation analysis between different studied parameters was demonstrated in (Table 6).

**Table 6:** Tissue Expression Correlations of Different Parameters in Studied Groups

	Correlation Coefficient, r	*P* value
**Tissue Fas versus** **Tissue FasL**	0.753	> 0.001
**Tissue Fas versus sFas, pg/ml**	- 0.321	> 0.05
**Tissue FasL versus sFas, pg/ml**	0.682	> 0.01

Electron Microscopic and Immunoelectron Microscopic examinations of Agarose Cell-Block

**Figure 3:**
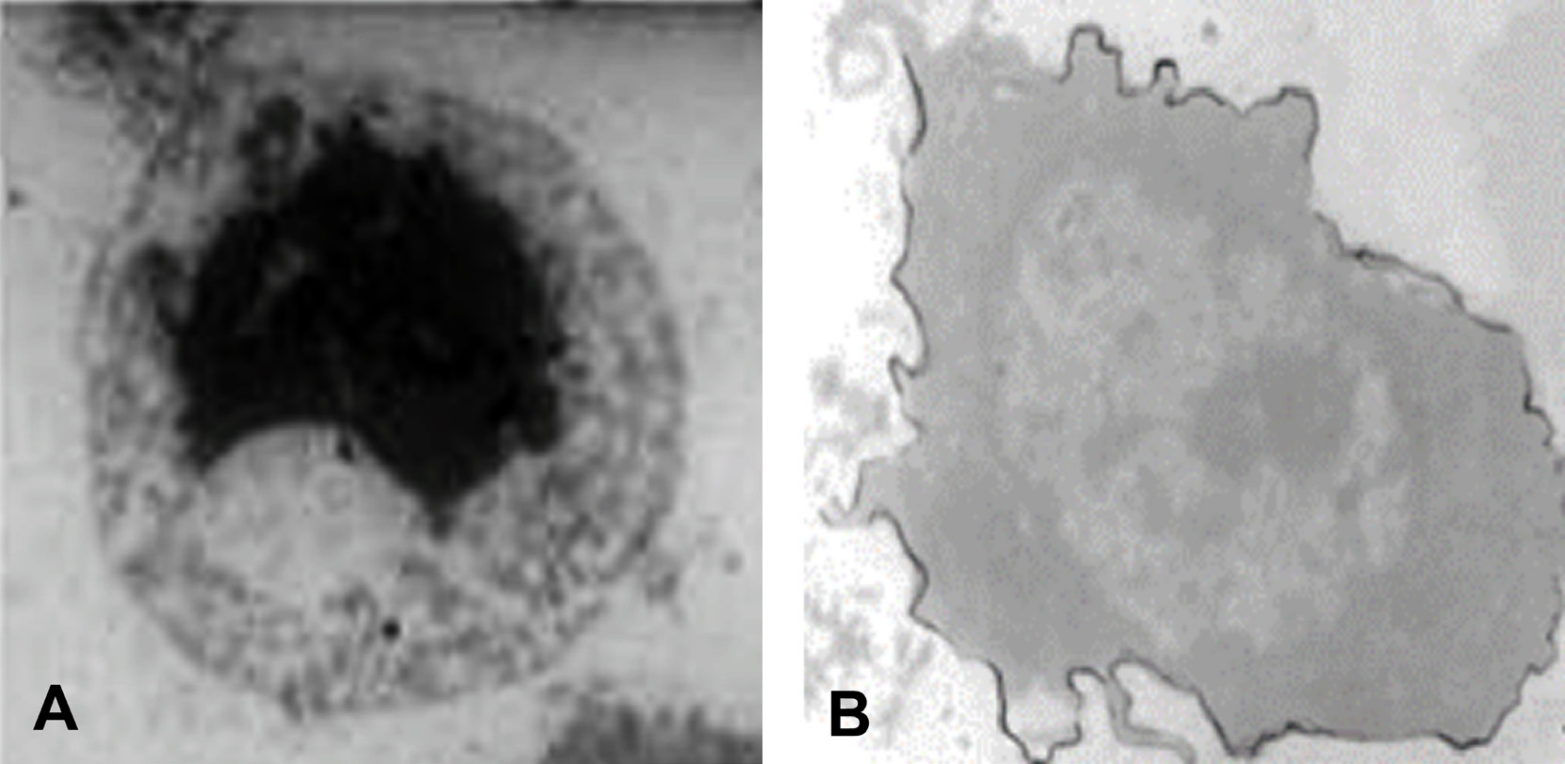
A) IEM photomicrograph of a immuno-peroxidase-labeled PB lymphocyte, showing over-expression of FasR alongside the cell membrane (uncontrasted section; X 1800). B) Peripheral blood lymphocyte in a patient with HCC, showing clumped nuclear chromatin (N) and vacuolated cytoplasm (X 2300).

There was significant over-expression of Fas on separated lymphocytes associated with a higher frequency of apoptotic cell death detected by EM examination (Figure 3).

## 5. Discussion

Apoptosis is central for the control and elimination of viral infections. In chronic HCV infection, the disease persists in the majority of patients despite enhanced hepatocyte apoptosis and up-regulation of death inducing ligands (21). The impact of apoptosis in chronic HCV infection is not well understood. It may be harmful by triggering liver fibrosis, or essential in interferon-induced HCV elimination (22). In addition, escape from the immune surveillance may play an important role in tumor outgrowth and metastasis. The Fas receptor/ligand system, including soluble forms, is the most important apoptosis initiator in the liver (23). Dysregulation of this pathway may contribute to abnormal cell proliferation and cell death (24) and is regarded as one of the mechanisms that prevents the immune system to reject tumor cells (13). In this study, the immune histochemical expression of Fas and FasL was determined using specific monoclonal antibodies. In normal liver, Fas was faintly expressed on cytoplasm and membranes of hepatocytes, while FasL was negative. This result was consistent with those of Leithauser et al. (25) and Roskams et al. (26). Data also showed the increased expression of Fas receptors and ligands in hepatocytes in liver specimens from CHC patients with and without cirrhosis, compared to the controls. Hepatic Fas expression was found to be elevated in chronic hepatitis B (27), chronic hepatitis C (28, 29), and acute liver failure (30). Moreover, a progressive Fas and FasL increasing from CH to cirrhosis was also observed (31). Our results suggest that “suicide” and “fratricide” mechanisms proposed by Galle et al. (30) in the study of alcoholic liver cirrhosis may also work in liver cirrhosis after viral hepatitis C, and may explain the mechanism by which apoptosis might be involved in the pathogenesis of liver cirrhosis. “Fratricide” is induced by ligation of Fas receptor and FasL expressed on the surface of adjacent cells, while “suicide” is induced by binding of FasL expressed by hepatocytes to Fas on the surface of the same cells. According to METAVIR activity score system, the up-regulation of hepatic Fas expression demonstrated in our study in CHC patients was in accordance with the severity of liver inflammation. Similar results were obtained by Pelli et al. (32). The histological activity index (HAI) revealed more expression of Fas antigen in liver tissues with active inflammation than in those without active inflammation (33). When HCV-specific T cells migrate into hepatocytes and recognize the viral antigen via T cell receptors, they become activated and express FasL that can transduce the apoptotic death signal to Fas-bearing hepatocytes (30, 32). This was proved in our study by prominent expression of FasL protein in infiltrating mononuclear cells in portal area and hepatic sinusoids .Thus, the Fas system plays an important role in liver cell injury by HCV infection. Our results also demonstrate that the expression of Fas and FasL was increased in the course of liver fibrosis. Several studies have demonstrated the increased expression of Fas and FasL in the course of liver fibrosis, which is the most serious consequence of chronic liver injury (28, 34, 35). Experimentally, the up- regulation of Fas expression occurs with stimulation by gamma interferon released from T cells (36). Cytokines and transforming growth factor-beta1 released from Kupfer cells and infiltrating T lymphocytes were also suggested to be the causes of hepatic fibrogenesis accompanied with hepatocellular necrosis and inflammation (28). Because hepatic fibrosis is associated with a high incidence of HCC, it was speculated that Fas expression might involve in the incidence of HCC (37, 38). In HCC liver biopsies, FasL showed cytoplasmic positivity in hepatocytes in areas of interface hepatitis. Strong expression of Fas as well as FasL in the hepatocytes immediately adjacent to HCC was a constant finding. Within the HCC biopsies, FasL expression was variable, but present only in a minority of cells. Fas varied from a diffuse honeycomb pattern to focal positivity in occasional cells (26). In recent years, there is accumulating evidence showing that sFas plays an important role in modulation of apoptosis (29). sFas was capable of inhibiting hepatic apoptosis by binding to FasL or anti-Fas antibodies in competition with membrane-bound Fas. SFas protein, composed of an extra cellular region of Fas receptor and Fc sequence of human IgG, inhibited the activity of Cytotoxic T lynphocytes (CTLs) in a dose-dependent manner (39). Midis et al. (40) reported that patients with non-haematopoietic malignancies exhibit elevated sFas levels compared to normal controls, and that sFas could be synthesized and released in the culture supernatants of human solid tumor explants. They also found that the relative elevation of sFas levels in non-haematopoietic cancer patients might be in consistence with both disease stage and tumor burden. Many researchers reported that serum sFas levels in patients with HCC were significantly higher than that in healthy adults (28, 39). Judo et al. (40) also reported that sFas levels was related to the number of tumor nodules, but not to the size of solitary nodule. In patients with solitary HCC nodule, serum sFas levels fell rapidly after surgical resection and went undetectable in one week. This evidence suggested that sFas was generated by tumor cells, or at least it was tumor-related. sFas mediated a prereceptorial resistance of Fas-expressing hepatocytes by antagonizing FasL killing effect of infiltrating CTLs. Thus, Fas-bearing tumor cells were saved by sFas from the fate of apoptosis and escape from the immune surveillance. Our data revealed significantly higher serum sFas levels in HCC patients than those in healthy adults, but showed no significant difference between the levels in HCC and cirrhotic patients. This phenomenon may be due to relatively high expression of FasL in HCC cells; sFas cleaved by metalloproteinases from trans-membrane domain might be capable of binding to sFas, thus accuracy of the measurement was influenced. A linear correspondence between liver tissue expression and serum levels of sFas was also detected. In conclusion, apoptosis and Fas system were significantly involved in the process of converting liver cirrhosis into hepatocellular carcinoma. Down-regulation of Fas expression, up-regulation of FasL expression in hepatocytes, and elevation of serum sFas levels were important in tumor evasion from immune surveillance and in hepatic carcinogenesis, and therefore, warranted and drawn the attention to the use of these components of the Fas system as attractive targets for anticancer therapy. In addition, the linear correspondence between liver tissue expression of Fas and its serum levels suggests that they could be considered as predictive markers for tumorigenesis in HCC.
